# West Nile fever in Czechland.

**DOI:** 10.3201/eid0504.990430

**Published:** 1999

**Authors:** Z Hubálek, J Halouzka, Z Juricová


### References

1. World Health Organization. Report of the meeting of the ad hoc committee on Orthopox virus infections. Department of Communicable Disease Surveillance and Response. WHO, 14-15 January 1999.
2. Cockburn WC, Cross RM, Downie AW, Dumbell KR, Kaplan C, Mclean D, et al. Laboratory and vaccination studies with dried smallpox vaccines. Bull World Health Organ 1957;16:63-77.

### West Nile Fever in Czechland

**To the Editor:** After heavy rains in July 1997, extensive floods occurred along the Morava River, Czech Republic. Populations of *Aedes* mosquitoes increased rapidly in the flooded areas, prompting surveillance for mosquito-borne virus infections in the Breclav area, South Moravia. We collected 11,334 female mosquitoes (9,100 *Aedes vexans,* 917 *Ae. cinereus,* 11 *Ae. cantans,* 1,074 *Ae. sticticus*, and 232 *Culex p. pipiens*) from July through September 1997 and tested them for virus in 117 monospecific pools by intracranial inoculation of suckling mice. Seven virus isolates were obtained and identified by complement-fixation and neutralization tests. Six isolates (five from *Ae. vexans*, one from *Ae. cinereus*) were identified as the bunyavirus Tahyna, California serogroup, and one (strain 97-103 from 57 *C. p. pipiens* collected at Lanzhot, 48°40'N, 16°56'E, on September 17) was identified as the flavivirus West Nile (1). A crossed comparison of 97-103 and topotype Eg-101 (2) West Nile virus strains and their antisera (prepared in mice by three intraperitoneal doses at weekly intervals) by plaque reduction neutralization (PRN) on XTC-2 cells (3,4) showed their antigenic relationships: reciprocal titers of homologous/heterologous sera were 512/512 in Eg-101 and 512/64 in 97-103. Strain 97-103 has lower virulence than Eg-101 in that it does not kill adult ICR mice and may represent a subtype of West Nile virus.

Blood samples were obtained from 619 persons seeking treatment at hospital and outpatient clinics in the Breclav area from June 23 through September 29, 1997. Sera were inactivated at 56°C for 30 minutes, diluted 1:8, and assayed by PRN for antibodies against *c.* 30 plaque-forming units (PFU) per well of West Nile virus strains Eg-101 and 97-103. All sera causing 90% reduction of PFU at 1:8 dilution were titrated, and the highest serum dilution showing 50% PFU reduction was regarded as the titer. Antibodies neutralizing West Nile virus were detected in 13 (2.1%) persons: 2.8% of 179 male and 1.8% of 440 female. Persons with detectable West Nile virus antibody were questioned about their health history during the previous 5 years, and their medical records were reviewed; none recalled having had tickborne encephalitis (Central-European encephalitis [CEE] virus is the only other flavivirus present in Czechland) or having been vaccinated against CEE or yellow fever virus. Titers of PRN antibodies to CEEV were all below 16. Two of the seropositive persons had traveled abroad during the last 5 years: one to Croatia in 1996, and one to South Australia during 1951 to 1994.

Paired serum samples were obtained from 72 of the 619 persons examined. A significant increase (≥4 times) in antibody titer against West Nile virus between the first (acute-phase) and second (convalescent-phase) samples was detected four times: in 2 of 41 young persons (≤16 years of age) and in 2 of 31 adults (>16 years of age). Among the four seroconverting persons, only the two children had clinical symptoms compatible with West Nile fever. A 9-year-old boy had fever (39°C) for 4 days, sore throat, headache, muscle ache, pronounced fatigue, and nausea lasting approximately 6 days, with recovery after 13 days. Neutralizing antibodies to West Nile virus, Eg-101 and 97-103, were 64 and 32 on July 22 and 512 and 256 on August 4, respectively. A 9-year-old girl had fever (38°C-39°C) for 3 days, sore throat, headache, muscle ache, pronounced fatigue, nausea, vomiting, maculopapular rash (including flushed face), and slightly enlarged inguinal lymph nodes. The illness lasted approximately 7 days, with complete recovery after 17 days. Neutralizing antibodies to West Nile virus, Eg-101 and 97-103, were 64 and 32 on August 6 and 256 and 128 on August 20, respectively. Of the remaining nine seropositive persons lacking paired serum samples, one had severe headache, muscle ache, prolonged fatigue, nausea, pain on eye movement, maculopapular rash, and insomnia in summer of 1997. Two other persons had had "summer fever" (sore throat and lymphadenitis; headache with pain on eye movement) in 1997. The other persons who seroconverted did not report any substantial illness. In total, clinical symptoms in five persons are compatible with West Nile fever.

These are the first reported human cases of West Nile fever in Central Europe (5); an extensive outbreak occurred in Romania in 1996, with approximately 500 patients hospitalized and a 4% to 8% fatality rate (6,7). West Nile virus should be viewed as a potential agent of local sporadic cases, clusters, or outbreaks, even in temperate Europe. Environmental factors (including human activities) that enhance vector population densities (heavy rains followed by floods, irrigation, higher than usual temperatures due to global warming) might produce an increased incidence of West Nile fever and other new or reemerging mosquito-borne diseases. Surveillance for West Nile fever should monitor population density and infection rate of principal vectors, antibodies in vertebrates and exposed human groups, and routine diagnosis of human infections.

**Zdenek Hubálek, Jirí Halouzka, and Zina Juricová**
Institute of Vertebrate Biology, Academy of Sciences, Brno, Czech Republic

### References

1. Hubálek Z, Halouzka J, Juricová Z, Šebesta O. First isolation of mosquito-borne West-Nile virus in the Czech Republic. Acta Virol 1998;42:119-20.
2. Melnick JL, Paul JR, Riordan JF, Barnett VHH, Goldblum N, Zabin E. Isolation from human sera in Egypt of a virus apparently identical to West Nile virus. Proc Soc Exp Biol Med 1951;77:661-5.
3. de Madrid AT, Porterfield JS. The flaviviruses (group B arboviruses): a cross-neutralization study. J Gen Virol 1974;23:91-6.
4. Leake CJ, Varma MGR, Pudney M. Cytopathic effect and plaque formation by arboviruses in a continuous cell line (XTC-2) from the toad *Xenopus laevis*. J Gen Virol 1977;35:335-9.
5. Hubálek Z, Halouzka J. Arthropod-borne viruses of vertebrates in Europe. Acta Scientiarum Naturalium Academiae Scientiarum Bohemicae Brno 1996;30, no. 4-5:1-95.
6. Le Guenno B, Bougermouh A, Azzam T, Bouakaz R. West Nile: a deadly virus? Lancet 1996;348:1315.
7. Tsai TF, Popovici F, Cernescu C, Campbell GL, Nedelcu NI. West Nile encephalitis epidemic in southeastern Romania. Lancet 1998;352:767-71.

### Ofloxacin-Resistant *Vibrio cholerae* O139 in Hong Kong

**To the Editor:** Unexpected outbreaks of cholera occurred in many areas of the world in 1997-98, partly because of weather changes associated with the El Niño phenomenon (1). Outbreaks caused by antibiotic-resistant *Vibrio cholerae* O1 and O139 have been documented in the Indian subcontinent (2-4), Africa (5), and Ukraine (6).

In Hong Kong, nonduplicate bacterial strains of *V. cholerae* O1 and O139 isolated from patients and environmental sources and received in the Public Health Laboratory between January 1, 1993, and June 30, 1998, were identified by conventional biochemical tests (7,8) and API 20E (bioMerieux, France); serotyped by slide agglutination with polyvalent O1 and mono-specific Inaba and Ogawa antisera (Murex, Dartford, United Kingdom); and checked with O139 antiserum (Denka Seiken, Tokyo, Japan). Biotyped and antibiotic susceptibilities were determined by the Kirby-Bauer disk-diffusion assay (8-10). Antibiotics tested included chloramphenicol and tetracycline (from 1993 to 1996) and ofloxacin (added in routine testing from 1997). *V. cholerae* isolates available for further study were tested with the standard broth microdilution method (11) to measure minimum inhibitory concentrations (MICs) of susceptibilities to chloramphenicol, tetracycline, and ofloxacin.

No antibiotic resistance was seen in *V. cholerae* isolates in testing conducted from 1969 to 1995. The first *V. cholerae* isolate with reduced susceptibility to chloramphenicol but sensitive to tetracycline was encountered in Hong Kong in 1996. This O1 El Tor Ogawa strain was imported from Nepal. Since then, more O1 strains were isolated that exhibited reduced antibiotic susceptibilities to chloramphenicol and tetracycline but not to ofloxacin (12). In May 1998, seven *V. cholerae* O139 strains were isolated that displayed patterns of antibiotic susceptibilities strikingly different from those of O1 isolates; the former were all sensitive to tetracycline but showed reduced susceptibilities to chloramphenicol and ofloxacin. All *V. cholerae* O1 strains tested have been susceptible to ofloxacin; O1 isolates falling into intermediate categories for chloramphenicol and tetracycline susceptibilities (31% and 27.6%, respectively) were common.

The first isolate of *V. cholerae* O139 in Hong Kong came from the imported case of a patient who had traveled to other provinces of China (13,14). Isolation of O139 continued sporadically since then, with six cases between 1993 and the

